# Safety and effects of two mistletoe preparations on production of Interleukin-6 and other immune parameters - a placebo controlled clinical trial in healthy subjects

**DOI:** 10.1186/1472-6882-11-116

**Published:** 2011-11-24

**Authors:** Roman Huber, Holger Lüdtke, Johannes Wieber, Christiane Beckmann

**Affiliations:** 1 Uni-Zentrum Naturheilkunde, University Medical Center Freiburg, Germany; 2 DatInf GmbH, Tübingen, Germany; 3 WALA Heilmittel GmbH, Bad Boll, Germany

## Abstract

**Background:**

In Germany, Iscucin^® ^Populi (IP), a preparation from mistletoe growing on the poplar tree, is used in cancer therapy while Viscum Mali e planta tota (VM), a preparation from mistletoe growing on the apple tree, is used in patients with osteoarthritis. Since mistletoe preparations are suspected to induce production of potentially tumor promoting cytokines like interleukin (IL)-6, further studies on the immunological effects are of interest.

**Methods:**

In this 3-armed randomized, double blind clinical trial healthy volunteers received increasing doses of either IP (strength F, 0.0125%, G, 0.25% and H, 5%, each for 4 weeks), or VM (1:1000 [D3], 1:100 [D2] and 2% each for 4 weeks) or placebo (isotonic solution) subcutaneously twice per week over a period of 12 weeks. Physical examination was performed weekly. Routine laboratory parameters and immunological parameters (C-reactive protein (CRP), differential blood count, lymphocyte subsets, immunoglobulins, IL-6 and tumor necrosis factor (TNF)-α) were analysed every 4 weeks.

**Results:**

71 subjects were included in the study (IP = 30, VM = 21, placebo = 20) of whom 69 concluded it according to protocol. Application of IP strengths G and H caused strong local reactions at the site of injection. In parallel, a distinct eosinophilia (p < 0.001 compared to placebo) occurred. Furthermore, application of all IP concentrations resulted in an increase of CD4 cell counts (p < 0.05) compared to placebo. Stimulation of IL-6 production, CRP or relevant deviations in other laboratory parameters were not observed. Because of local reactions, IP strengths G and H were considered less tolerable than placebo. VM 2% was slightly less tolerable than placebo, caused only mild local reactions and an only small increase in eosinophile counts.

**Conclusion:**

Treatment with IP results in eosinophilia and an increase of CD4 cells but not in an increase of IL-6 or CRP. No safety concerns regarding the two mistletoe preparations have been raised by this study. EudraCT-Number 2007-002166-35.

**Trial registration:**

ClinicalTrials.gov: NCT01378702

## Background

Mistletoe preparations are widely used among cancer patients in Europe [1,2]. Increasing evidence exists, that mistletoe preparations containing mistletoe lectins (ML) have beneficial effects on the quality of life of cancer patients during chemo- or radiation therapy [3]. Because they can stimulate the production of interleukin (IL)-6 in periphereal mononuclear cells and lymphoma cells *in vitro *it has been hypothesized, that they promote tumor enhancement of related malignancies [4,5]. IL-6 is an important growth factor for lymphoma and multiple myeloma cells in vitro and in vivo [6]. However, *in vitro *experiments incubating mistletoe extracts with lymphoma and multiple myeloma cells have shown dose dependent apoptosis mediated growth inhibition and no stimulation [7-9]. Furthermore, a retrospective study of 700 lymphoma patients who received mistletoe extracts suggested this therapy to be beneficial and revealed no hint for tumor promoting effects [10]. Nevertheless, the German regulatory authority (BfArM, Federal Institute for Drugs and Medical Devices) demanded further studies to prove safety.

Iscucin^® ^Populi (IP) and Viscum Mali e planta tota (VM) are two mistletoe preparations that have been used in anthroposophical medicine for decades in Germany [11-13]. IP is registered for complementary cancer therapy [11,12]. VM is used to reduce pain and improve mobility in patients with arthropathies [13]. The study was performed in order to fulfil the requirements of the BfArM.

Because cancer patients are a heterogeneous collective in whom effects of mistletoe treatment on immune function and safety are compromised by the underlying disease and/or conventional therapies healthy volunteers were recruited for the clinical trial. The decisive advantage of this setup is that healthy volunteers are a comparably homogenous collective with respect to their immune system and that concomitant medication can be excluded.

## Methods

The trial was performed as a multiple dose, randomized, placebo-controlled, double blind, monocenter dose escalation clinical trial at University Medical Center Freiburg. The primary outcome criterion was to describe the safety of IP and VM (adverse events: changes in vital signs, physical examination and routine laboratory). Secondary endpoints were the effects of IP and VM on the immune system (interleukin (IL)-6, C-reactive protein, differential blood count, lymphocyte subsets (total lymphocytes (CD3), CD4 cells, CD8 cells, B (CD19) cells, natural killer (CD56) cells), tumor necrosis factor (TNF)-α, immunoglobulins A, G, M, mistletoe antibodies) and estimation of tolerability on a four point rating scale (bad, moderate, good, excellent)

Healthy male and female volunteers between 18-45 years of age with a body mass index (BMI) between 18.5-28 kg/m^2^, normal blood pressure, normal physical examination and normal routine laboratory values (haematology, coagulation parameters, creatinine, urea, uric acid, electrolytes, creatine kinase, liver enzymes, lactate dehydrogenase, bilirubin, total protein, albumin, alpha amylase, lipids, glucose), negative Hepatitis B, C and HIV serology, negative urine screen for drug abuse and no ethanol in blood were included in the study. Exclusion criteria were presence or sequelae of any clinically significant disease, drug abuse, smoking more than 20 cigarettes a day, any medication in the week before inclusion, participation in another clinical trial in the 3 preceding months, previous therapy with mistletoe preparations, history of allergy to medicinal products, donation of blood in the 3 preceding months, pregnancy or breast-feeding, absence of highly effective contraception and inability to understand the nature and extent of the trial.

Only volunteers who had given written informed consent and met all the eligibility criteria were included into the study. The study had a positive vote from the ethics committee of the Faculty of Medicine at the Albert-Ludwigs University of Freiburg (Germany) and was carried out in compliance with the principles of Good Clinical Practice and the Declaration of Helsinki.

At baseline the subjects were randomly assigned to IP, VM or placebo. The subjects injected increasing doses of either IP (strength F, 0.0125%, G, 0.25% and H, 5%, each for 4 weeks) or VM (1:1000 [D3], 1:100 [D2] and 2% each for 4 weeks) or placebo subcutaneously in the abdomen twice per week (interval 3 and 4 days) for a period of 12 weeks. One ampoule of IP strength H was regarded as the maximum tolerated dose, because it was expected to cause strong local reactions [14,15]. In case of strong local reactions dose reductions could be performed analogously to the procedure used in tumor patients in the sequence: ½ ampoule, ¼ ampoule, ⅛ ampoule, until subjective tolerability was achieved. 50% of the injections were administered under supervision of a study nurse or physician. The respective medication for the next 4 weeks was handed out at baseline, after 4 weeks and after 8 weeks. Clinical and safety controls (physical examination, inspection of the injection site) were performed weekly. Routine laboratory parameters (see above), immunological parameters (see above) and estimation of tolerability (bad, moderate, good, excellent) by the subjects were documented every 4 weeks always in the morning to the same time (± 1 hour) as baseline investigations. Mistletoe antibodies were determined in week 12. Safety and immunological parameters were checked again in a follow up visit 4 weeks after the last injection, The stepwise increase in dosage was performed because we hypothesized an increase of side effects and potential stimulation of the immune system with the dosage. According to this hypothesis, low dosages (strength F of IP, all dosages of VM) of the two mistletoe preparations should be well tolerable and cause only mild immunological changes as described in a similar study investigating a different mistletoe preparation [16]. Because of the high content of cytotoxic mistletoe lectin in IP strength G and H, we expected higher rates of side effects in the groups receiving IP strength G and H.

### Study medication

Iscucin^® ^Populi (IP) is an aqueous extract from mistletoes growing on poplar trees. The dried plant is extracted with isotonic solution over a 14 day period without fermentation. One ampoule (1 ml) of the concentration 1:20 (5%, strength H) contains 50 mg of the mother extract; concentration 1:400 (0.25%, strength G) contains 2.5 mg and concentration 1:8000 (0.0125%, strength F) 0.125 mg, respectively.

Viscum Mali e planta tota (VM) is an aqueous fermented extract from fresh mistletoes growing on apple trees. 1 ampoule (1 ml) of the concentration 1:1000 (third decimal, D3, according to the German homeopathic pharmacopoeia) contains 1 mg of the mother extract, concentration 1:100 (second decimal, D2) 10 mg and concentration 2:100 (2%) 20 mg. Placebo was the isotonic solution that is used for the preparation of IP and VM consisting of sodium chloride, sodium bicarbonate and water for injection.

### Laboratory parameters

Routine laboratory parameters were analyzed within less than 1 h of taking the probes by standard methods at the central laboratory of University Medical Center Freiburg according to Good Laboratory Practice (GLP). C-reactive protein (CRP) was determined using a turbidimetric, latex enhanced immunoassay. Lymphocyte subsets were analyzed by flow cytometry (FACS Calibur, Becton Dickinson, Heidelberg, Germany), using anti-CD3, CD4, CD8, CD19 and CD56 monoclonal antibodies (Becton Dickinson, Heidelberg, Germany).

For analysis of IL-6, TNF-α, mistletoe lectin- and viscotoxin antibodies blood was centrifugated at 3000 g/min for 5 min and sera were stored at -18°C at the clinical trial centre until analyses at the end of the study. IL-6 and TNF-α were determined by ELISA at the central laboratory of the University Medical Center Freiburg. Mistletoe antibodies were determined by ELISA as described in [17] at the Department of Internal Medicine II of Tübingen University Hospital, Germany.

All analyses were performed blinded.

### Statistics

The primary analysis was done on the safety population, defined as all subjects who had received at least one ampoule of the study medication and was identical to the intention to treat analysis. All primary analysis was descriptive. For categorical variables number and percentage were calculated, for continuous variables mean, standard deviation, median, minimum and maximum were presented. Courses of laboratory parameters were analysed by generalised estimation equations (GEE), hereby modeling treatment group and visit number as fixed cofactors, the respective baseline value as a linear covariate and the patient identification as the repeated measurement factor. The correlation structure between the visits was assumed to be first-order autoregressive. All estimated group differences (IP versus placebo and VM versus placebo) were based on these GEE models. Tolerability was analysed by exact two-sided Wilcoxon-Mann-Withney tests. The significance level was set to 0.05. As usual for safety studies, no correction for multiple testing was performed.

Randomisation was performed by the biometrical centre (DatInf GmbH, Tübingen, Germany) using block randomisation with variable block size. Allocation to the groups was concealed. Blinding was performed by the manufacturer. All ampoules were identically labelled and packed in boxes with identical appearance. All analyses and data management were performed blinded.

As this was a modified phase I dose escalation study 20 subjects per group have been planned in analogy to similar studies with other mistletoe preparations [16,18]. Because a higher drop-out rate and greater number of side effects were expected in the IP group due to high concentrations of especially mistletoe lectins (ML), 30 subjects were chosen for this group to make sure that at least 20 could be analyzed.

## Results

**Figure 1:**
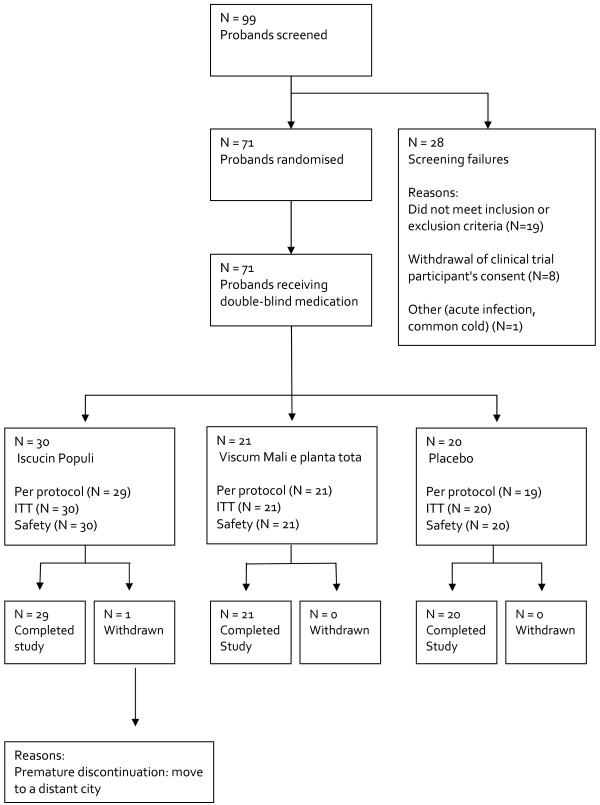
**Flow chart of the study**.

99 subjects were screened and 71 were randomised. 27 subjects were either screening failures or withdrew their consent; one subject was included but not randomised because of a common cold at baseline (visit 1, Figure 1).

70 of 71 subjects concluded the study, one in the VM group stopped prematurely (after the first injection), because she moved to a distant city 800 km from the study centre. One subject from the placebo group was excluded from the per protocol analysis because of major protocol violations. Therefore, the per protocol analysis comprised 69 subjects and the intention to treat analysis 71 subjects. Table 1 shows the characteristics of the subjects. There were no significant differences between the groups at baseline.

**Table 1:** Characteristics of the subjects (percentage or mean ± standard deviation)

	IP (n = 30)	VM (n = 21)	Placebo (n = 20)
Male/female (%)	40/60	29/71	34/66
Age (years)	29 ± 7	30 ± 7	29 ± 7
BMI (kg/m^2^)	22 ± 3	22 ± 2	22 ± 3
Smokers (%)	17	19	30

Analysis of the immunological parameters revealed a strong and significant eosinophilia following injection of IP compared to baseline and placebo (Table 2, p = 0.006 in week 4, p < 0.001 in week 8 and 12). At follow up eosinophil counts were back to baseline values. VM resulted in an only small but also significant increase of eosinophil counts (Table 2, p = 0.019 and p = 0.009 compared to placebo in week 8 and 12, respectively). The increase in eosinophil counts in the IP group was paralleled by the number of total leukocytes (p = 0.038 and p = 0.043 in week 8 and 12 respectively, Table 2) compared to placebo. Also, the absolute number of lymphocytes was slightly higher in the IP group (p = 0.042, 0.029 and 0.044 at week 4, 8 and 12, respectively), which could be related to a slight increase of the absolute number of CD4 cells (p = 0.017, p = 0.009, p = 0.010 and p = 0.031 in week 4, 8, 12 and 16 respectively) and CD8 cells (p = 0.038 in week 12, Table 2).

**Table 2:** Means ± standard deviation of immunological parameters before, during and after exposition with Iscucin^® ^Populi (IP), Viscum Mali e planta tota (VM) and placebo (Pl) in the ITT population, nd = not done.

	Group	Baseline	Exposure	Exposure	Exposure	Follow-up
			Week 4	Week 8	Week 12	Week 16
Leukocytes (1000/μl)	IP	6.2 ± 2.0	5.9 ± 1.2	7.2 ± 2.0 *	6.7 ± 1.6 *	5.8 ± 1.5
	VM	5.7 ± 1.4	5.8 ± 1.2	6.0 ± 1.2	5.6 ± 1.4	5.7 ± 1.0
	Pl	6.4 ± 1.7	6.2 ± 1.6	6.5 ± 1.7	6.1 ± 1.7	6.5 ± 1.8

Eosinophils (1000/μl)	IP	0.1 ± 0.0	0.2 ± 0.1**	1.1. ± 0.7***	0.8 ± 0.5***	0.2 ± 0.1
	VM	0.1 ± 0.0	0.1 ± 0.0	0.2 ± 0.0*	0.2 ± 0.0**	0.2 ± 0.1
	Pl	0.2 ± 0.1	0.2 ± 0.1	0.2 ± 0.1	0.2 ± 0.1	0.2 ± 0.1

Neutrophils (1000/μl)	IP	3.8 ± 1.7	3.3 ± 0.9	3.7 ± 1.4	3.5 ± 1.3	3.4 ± 1.3 *
	VM	3.3 ± 1.1	3.3 ± 0.9	3.5 ± 1.2	3.2 ± 1.1	3.3 ± 0.7
	Pl	3.9 ± 1.5	3.8 ± 1.3	4.0 ± 1.4	3.8 ± 1.6	4.1 ± 1.5

Lymphocytes (1000/μl)	IP	1.9 ± 0.5	2.0 ± 0.4*	2.1 ± 0.5*	2.0 ± 0.5*	1.9 ± 0.5
	VM	1.9 ± 0.6	2.0 ± 0.5	1.9 ± 0.5	1.9 ± 0.5	1.9 ± 0.6
	Pl	1.9 ± 0.3	1.9 ± 0.4	1.9 ± 0.4	1.8 ± 0.3	1.8 ± 0.3

Monocytes (1000/μl)	IP	0.4 ± 0.1	0.3 ± 0.0	0.4 ± 0.1	0.4 ± 0.1	0.3 ± 0.0**
	VM	0.3 ± 0.0	0.3 ± 0.0	0.4 ± 0.1	0.3 ± 0.1	0.3 ± 0.1
	Pl	0.3 ± 0.1	0.3 ± 0.0	0.3 ± 0.1	0.3 ± 0.1	0.4 ± 0.1

Natural killer cells (1000/μl)	IP	0.3 ± 0.1	0.3 ± 0.1	0.3 ± 0.1	0.3 ± 0.1	0.2 ± 0.1
	VM	0.2 ± 0.1	0.2 ± 0.1	0.2 ± 0.0	0.2 ± 0.1	0.2 ± 0.1
	Pl	0.3 ± 0.0	0.3 ± 0.0	0.3 ± 0.1	0.2 ± 0.0	0.2 ± 0.0

CD4 cells (1000/μl)	IP	0.8 ± 0.3	0.9 ± 0.3*	0.9 ± 0.3*	0.9 ± 0.3*	0.9 ± 0.3*
	VM	0.8 ± 0.2	0.9 ± 0.2	0.8 ± 0.2	0.9 ± 0.2	0.8 ± 0.3
	Pl	0.9 ± 0.2	0.8 ± 0.1	0.8 ± 0.2	0.8 ± 0.1	0.8 ± 0.2

CD8 cells (1000/μl)	IP	0.4 ± 0.1	0.5 ± 0.1	0.5 ± 0.1	0.5 ± 0.1*	0.5 ± 0.1
	VM	0.5 ± 0.2	0.6 ± 0.2	0.5 ± 0.2	0.5 ± 0.1	0.5 ± 0.1
	Pl	0.5 ± 0.1	0.5 ± 0.1	0.5 ± 0.1	0.5 ± 0.1	0.5 ± 0.1

Ratio T4/T8	IP	2.0 ± 0.7	2.0 ± 0.7*	2.0 ± 0.7	2.0 ± 0.7	2.0 ± 0.7
	VM	1.7 ± 0.7	1.8 ± 0.7	1.8 ± 0.7	1.8 ± 0.7	1.8 ± 0.6
	Pl	1.8 ± 0.5	1.8 ± 0.5	1.8 ± 0.5	1.9 ± 0.6	1.8 ± 0.5

B-cells (1000/μl)	IP	0.2 ± 0.1	0.2 ± 0.1	0.2 ± 0.1	0.3 ± 0.1	0.2 ± 0.1
	VM	0.2 ± 0.0	0.2 ± 0.0	0.2 ± 0.1	0.2 ± 0.0	0.2 ± 0.0
	Pl	0.2 ± 0.0	0.2 ± 0.0	0.2 ± 0.0	0.2 ± 0.0	0.2 ± 0.1

Immunoglobulin A (g/l)	IP	1.9 ± 0.7	1.8 ± 0.7	1.8 ± 0.7	1.8 ± 0.7	1.8 ± 0.7
	VM	2.2 ± 0.9	2.2 ± 0.8	2.2 ± 0.9	2.2 ± 0.9	2.2 ± 0.8
	Pl	1.9 ± 0.7	1.9 ± 0.7	1.9 ± 0.7	1.9 ± 0.7	1.8 ± 0.7

Immunoglobulin G (g/l)	IP	11.3 ± 2.4	11.1 ± 2.3	11.0 ± 2.1	11.1 ± 2.2	11.1 ± 2.4
	VM	11.1 ± 1.8	11.2 ± 1.7	11.1 ± 1.9	11.1 ± 1.7	11.0 ± 1.7
	Pl	11.0 ± 2.4	11.0 ± 2.3	11.0 ± 2.4	11.0 ± 2.3	11.0 ± 2.4

Immunoglobulin M (g/l)	IP	1.3 ± 0.5	1.3 ± 0.5	1.3 ± 0.5	1.3 ± 0.5*	1.3 ± 0.6*
	VM	1.3 ± 0.6	1.3 ± 0.7**	1.3 ± 0.7	1.3 ± 0.6	1.3 ± 0.6
	Pl	1.2 ± 0.6	1.2 ± 0.6	1.2 ± 0.6	1.1 ± 0.6	1.2 ± 0.6

Interleukin-6 (pg/ml)	IP	0.8 ± 0.8	0.6 ± 0.3	0.9 ± 1.3	1.3 ± 1.7	0.7 ± 0.5
	VM	0.6 ± 0.4	0.5 ± 0.2	1.0 ± 1.2	0.6 ± 0.4	0.6 ± 0.4
	Pl	1.0 ± 0.7	0.8 ± 0.7	0.9 ± 0.8	1.0 ± 0.8	1.7 ± 3.0

C-reactive protein (mg/l)	IP	2.7 ± 4.4	1.8 ± 1.1	2.1 ± 1.4	3.6 ± 5.5	1.6 ± 1.2*
	VM	2.5 ± 3.0	1.8 ± 1.1	3.4 ± 3.5	1.6 ± 1.2**	1.8 ± 1.2*
	PL	4.6 ± 9.2	3.0 ± 3.4	3.0 ± 2.6	4.2 ± 3.4	4.3 ± 5.0

Tumor necrosis factor-alpha (ng/l)	IP	10 ± 3	10 ± 3	11 ± 3	12 ± 3 *	11 ± 3
	VM	10 ± 3	10 ± 3	11 ± 4	11 ± 4	11 ± 3
	Pl	10 ± 3	10 ± 2	11 ± 3	10 ± 2	11 ± 2

Mistletoe lectin antibodies (optical density)	IP	nd	nd	nd	971 ± 143***	nd
	VM	nd	nd	nd	51 ± 31	nd
	Pl	nd	nd	nd	33 ± 30	nd

Viscotoxin antibodies (optical density)	IP	nd	nd	nd	544 ± 289***	nd
	VM	nd	nd	nd	76 ± 98	nd
	Pl	nd	nd	nd	13 ± 9	nd

* p < 0.05 compared to placebo, ** p < 0.01 compared to placebo, *** p < 0.001 compared to placebo. Significant deviations are bold typed.

No differences between the groups were found for IL-6. C-reactive protein was lower in the IP group at follow up compared to the placebo group (p = 0.018), and lower in the VM group at 12 weeks treatment and follow up as compared to the placebo group (p = 0.002 and p = 0.027, respectively).

Immunoglobulin M (IgM) slightly increased in the IP group compared to the placebo group after 8 and 12 weeks treatment (p = 0.033 and 0.022, respectively) and was also slightly higher in the VM group than in the placebo group after 4 weeks treatment (p = 0.007). This increase could, at least in the IP group, possibly be related to the mistletoe preparations, because IgM also increased slightly in comparison to baseline, and because antibody production, measured in week 12, was stimulated in the IP group. (compared to placebo p < 0.001 for ML- and viscotoxin antibodies). In the VM-group viscotoxin and ML-antibodies were not significantly different to the placebo group. TNF-α was at week 12 slightly higher in the IP group than in the placebo group (p = 0.010, Table 2). During exposure, no differences in absolute neutrophil counts, the number of natural killer cells, monocytes, T-suppressor cells, B-cells, immunoglobulins A and G were found for either IP or VM compared to placebo.

Application of IP strengths G (0.25%] and H (5%) caused local reactions (LR) at the site of injection in almost all subjects belonging to the IP-group (93%, Table 3). LR were strong, with a mean diameter of 2 cm in week 4, 6.1 cm in week 8, and 6.2 cm in week 12. Maximum diameter of redness occurred in week 6 (8.8 cm). Because of the strong, in part subjectively not tolerated local reactions, dose adjustments had to be performed for IP strengths G and H in line with the protocol and analogous to procedure in tumor patients [15]. The total number of ampoules injected was lower in the IP group compared to placebo (Table 3). Subsequently, tolerability of IP strengths G and H was estimated to be worse than placebo (p < 0.0001, Table 3). VM caused mild local reactions in 19% of the subjects at concentrations D2 and 2% (diameter up to a mean of 2 cm) and tolerability was also worse than placebo (p = 0.0207, exact two-sided Wilcoxon-Mann-Whitney test, Table 3). However, dose adjustments were not necessary (Table 3).

**Table 3:** Extent of exposure (maximum 24 ampoules = 100%), percentage of subjects with local reactions and tolerability.

	**Iscucin**^® ^**Populi**			Viscum Mali e planta tota			Placebo		
	(n = 30)			(n = 21)			(n = 20)		
	**0.0125%**	**0.25%**	**5%**	**1:1000**	**1:100**	**1:50**			
	**strength**	**strength**	**strength**	**D3**	**D2**	**2%**			
	**F**	**G**	**H**						
	**Week**	**Week**	**Week**	**Week**	**Week**	**Week**	**Week**	**Week**	**Week**
	**1-4**	**5-8**	**9-12**	**1-4**	**5-8**	**9-12**	**1-4**	**5-8**	**9-12**

Overall injected ampoules (%)	100.0	81.7	64.0	100.0	100.0	99.1	99.7	99.8	99.9
Local reactions %	17	93	93	0	19	19	0	0	0
Tolerability (%)		**	**			*			
Bad	0	**28**	**21**	0	0	**0**	0	0	0
Moderate	3	**38**	**45**	0	0	**0**	5	0	0
Good	21	**34**	**34**	5	14	**29**	0	0	0
Excellent	76	**0**	**0**	95	86	**71**	95	100	100

*p < 0.05 versus Placebo, **p < 0.001 versus Placebo. Significant deviations are bold typed.

In total, 183 adverse events (AE) were reported throughout the study, 95 in the IP group, 48 in the VM group and 40 in the placebo group. Local reactions were the most frequently reported AE and appeared in 28 from 30 subjects in the IP group, 5 from 21 subjects in the VM group and 0 from 20 subjects in the placebo group (Table 3). Also swollen lymph nodes were reported more frequently in the IP group (Table 4). A certain relation to the study medication was stated in 26% of the AE in the IP group, 10% in the VM group and 0% in the placebo group. Only local reactions and related side effects (swelling of local lymph nodes, localized itching, local pain of the abdominal wall, fever) had been classified as probably or certainly related to the study medication. Two serious AE occurred. One subject developed acute lumboischialgia (VM group) and one presented acute gastroenteritis (placebo group). Both needed hospitalisation. In both cases there was no relation to the study medication and both subjects continued the study without deblinding. Coded non serious AE (according to the Medical Dictionary for Regulatory Activities (MedDRA) terminology "low level term") in the three groups are shown in Table 4. Vital signs (body temperature, blood pressure and pulse rate, table 5) and physical findings (data not shown) were similar in the different groups. Laboratory parameters (except immunology) are shown in Table 6. For most parameters no differences could be found between the IP and the placebo group or between the VM and the placebo group. Neither could any differences at all be shown when the laboratory parameters were grouped into 'normal or without clinical relevance' and 'clinical significance with relevance'. This is supported by the fact that almost no group values exceeded the reference values.

**Table 4:** Adverse events as percentage of subjects per group during the three treatment blocks

	**Iscucin**^**® **^**Populi**			Viscum Mali e planta tota			Placebo		
	(n = 30)			(n = 21)			(n = 20)		
**Description of AE**	**0.0125%**	**0.25%**	**5%**	**1:1000**	**1:100**	**1:50**			
	**strength**	**strength**	**strength**	**D3**	**D2**	**2%**			
	**F**	**G**	**H**						
	**Week**	**Week**	**Week**	**Week**	**Week**	**Week**	**Week**	**Week**	**Week**
	**1-4**	**5-8**	**9-12**	**1-4**	**5-8**	**9-12**	**1-4**	**5-8**	**9-12**

Abdominal pain (%)	0	3	3	0	0	0	0	0	0
Common cold (%)	27	17	17	38	14	10	15	15	25
Diarrhea (%)	0	3	0	0	0	0	0	0	0
Dizziness (%)	0	3	0	0	0	0	5	0	0
Fatigue (%)	0	0	3	0	0	0	0	0	0
Fever (%)	0	3	0	0	0	0	0	0	0
Headache (%)	13	0	3	14	5	10	5	10	10
Nausea (%)	0	3	3	0	0	0	0	0	0
Swollen lymph nodes (%)	0	3	7	0	0	0	0	0	0
Vomiting (%)	3	3	0	0	0	0	0	0	0
Weakness (%)	0	0	0	5	0	0	5	0	0

**Table 5:** Vital signs (means ± standard deviation) in the ITT population.

	Group	Baseline	Exposure	Exposure	Exposure	Follow-up
			Week 4	Week 8	Week 12	Week 16
Body temperature	IP	36.4 ± 0.4	36.4 ± 0.4	36.3 ± 0.5	36.4 ± 0.4	36.5 ± 0.3
	VM	36.5 ± 0.4	36.2 ± 0.5	36.3 ± 0.5	36.3 ± 0.3	36.4 ± 0.3
	PL	36.3 ± 0.4	36.3 ± 0.3	36.4 ± 0.4	36.4 ± 0.3	36.5 ± 0.4
Systolic blood pressure	IP	120 ± 11	117 ± 11	114 ± 10	117 ± 11	112 ± 12
	VM	112 ± 10	114 ± 10	110 ± 11	111 ± 12	112 ± 13
	PL	112 ± 13	110 ± 11	113 ± 9	111 ± 13	112 ± 13
Heart rate	IP	76 ± 11	72 ± 7	74 ± 9	71 ± 9	72 ± 11
	VM	71 ± 9	74 ± 11	70 ± 8	68 ± 8	66 ± 9
	PL	74 ± 8	71 ± 9	72 ± 6	72 ± 9	74 ± 7

IP = Iscucin^® ^Populi, VM = Viscum Mali e planta tota, PL = placebo.

**Table 6:** Laboratory parameters (means ± standard deviation) in the ITT population.

	Group	Baseline	Exposure	Exposure	Exposure	Follow-up
			Week 4	Week 8	Week 12	Week 16
Hemoglobin (mg/dl)	IP	14.0 ± 1.0	14.0 ± 0.9	13.9 ± 1.0	14.0 ± 0.9	14.0 ± 0.9
	VM	13.6 ± 1.1	13.7 ± 1.0	13.7 ± 1.0	13.5 ± 1.1	13.6 ± 1.1
	PL	13.8 ± 0.9	13.8 ± 1.0	14.0 ± 1.1	13.8 ± 1.1	13.8 ± 1.1

Hematocrit (%)	IP	41.6 ± 2.6	41.7 ± 2.4	41.2 ± 2.6	41.4 ± 2.5	41.4 ± 2.1
	VM	40.7 ± 2.8	41.1 ± 2.7	40.7 ± 2.9	40.4 ± 3.1	40.5 ± 2.8
	PL	41.4 ± 2.1	41.4 ± 2.5	41.8 ± 2.7	41.3 ± 2.7	41.4 ± 2.8

Platelets (1000/μl)	IP	272 ± 52	259 ± 43	271 ± 44	256 ± 41*	250 ± 44
	VM	269 ± 46	272 ± 62	260 ± 59	251 ± 55*	256 ± 65
	PL	267 ± 56	265 ± 52	274 ± 66	271 ± 56	254 ± 52

Calcium (mmol/l)	IP	2.4 ± 0.0	2.4 ± 0.1	2.4 ± 0.0	2.4 ± 0.0	2.4 ± 0.0
	VM	2.3 ± 0.0	2.4 ± 0.1	2.4 ± 0.0	2.4 ± 0.0	2.4 ± 0.1
	PL	2.4 ± 0.0	2.4 ± 0.0	2.4 ± 0.0	2.4 ± 0.0	2.4 ± 0.1

Potassium (mmol/l)	IP	4.3 ± 0.2	4.3 ± 0.4	4.3 ± 0.3	4.2 ± 0.3	4.2 ± 0.2
	VM	4.3 ± 0.3	4.1 ± 0.2	4.2 ± 0.2	4.2 ± 0.2	4.3 ± 0.2
	PL	4.3 ± 0.2	4.2 ± 0.2	4.3 ± 0.3	4.3 ± 0.3	4.4 ± 0.4

Sodium (mmol/l)	IP	139 ± 2	139 ± 2	141 ± 2	140 ± 2	141 ± 2
	VM	139 ± 1	139 ± 2	141 ± 1	140 ± 2	140 ± 1
	PL	138 ± 2	139 ± 1	141 ± 2	140 ± 2	141 ± 2

Chloride (mmol/l)	IP	101 ± 2	102 ± 2	103 ± 2	102 ± 2	102 ± 2
	VM	102 ± 2	102 ± 2	104 ± 1	103 ± 2	103 ± 2
	PL	101 ± 2	102 ± 2	104 ± 2	103 ± 2	103 ± 2

Creatinine (mg/dl)	IP	0.8 ± 0.1	0.9 ± 0.1	0.8 ± 0.1	0.8 ± 0.1	0.8 ± 0.1
	VM	0.8 ± 0.1	0.8 ± 0.1	0.8 ± 0.1	0.8 ± 0.1	0.8 ± 0.1
	PL	0.8 ± 0.0	0.8 ± 0.1	0.8 ± 0.0	0.8 ± 0.1	0.8 ± 0.1

Urea (mg/dl)	IP	26 ± 6.	28 ± 7	28 ± 8*	27 ± 6	28 ± 6
	VM	26 ± 7	28 ± 8	28 ± 7*	29 ± 8	27 ± 7
	PL	30 ± 8	27 ± 6	26 ± 7	29 ± 8	30 ± 7

Uric acid (mg/dl)	IP	4.3 ± 1.0	4.5 ± 0.9	4.3 ± 1.1	4.3 ± 1.1	4.6 ± 1.1
	VM	4.2 ± 1.0	4.2 ± 0.8	4.1 ± 0.8	4.2 ± 0.9	4.2 ± 1.0
	PL	4.3 ± 0.9	4.3 ± 1.1	4.3 ± 1.1	4.5 ± 1.3	4.6 ± 1.1

AST (U/l)	IP	26 ± 8	25 ± 7	25 ± 9	25 ± 7	26 ± 7
	VM	25 ± 6	23 ± 5	37 ± 60	25 ± 6	25 ± 7
	PL	26 ± 7	24 ± 7	23 ± 6	23 ± 6	26 ± 10

ALT (U/l)	IP	20 ± 10	18 ± 7	16 ± 7	16 ± 7	17 ± 8
	VM	20 ± 9	17 ± 6**	20 ± 16	17 ± 8	17 ± 8
	PL	20 ± 10	21 ± 13	19 ± 16	18 ± 14	19 ± 12

Gamma GT (U/l)	IP	19 ± 9	18 ± 7	20 ± 9	19 ± 9	19 ± 9
	VM	15 ± 6	15 ± 6	15 ± 5	15 ± 5	14 ± 5
	PL	19 ± 10	20 ± 11	21 ± 12	20 ± 12	18 ± 12

Alkaline phosphatase (U/l)	IP	63 ± 20	61 ± 20	64 ± 19	64 ± 18*	63 ± 21
	VM	56 ± 17	56 ± 14	58 ±15	54 ± 12	54 ± 14
	PL	59 ± 14	59 ± 17	58 ±16	57 ± 14	58 ± 19

Bilirubin (mg/dl)	IP	0.6 ± 0.2	0.6 ± 0.1	0.6 ± 0.1	0.6 ± 0.1	0.6 ± 0.2
	VM	0.6 ± 0.3	0.7 ± 0.3	0.6 ± 0.3	0.7 ± 0.3*	0.6 ± 0.3
	PL	0.6 ± 0.3	0.6 ± 0.3	0.6 ± 0.5	0.6 ± 0.2	0.6 ± 0.3

Total protein (g/dl)	IP	7.7 ± 0.5	7.8 ± 0.4	7.7 ± 0.3	7.8 ± 0.4	7.8 ± 0.6
	VM	7.5 ± 0.3	7.8 ± 0.3	7.6 ± 0.3	7.6 ± 0.3	7.7 ± 0.3
	PL	7.6 ± 0.2	7.7 ± 0.2	7.7 ± 0.3	7.7 ± 0.3	7.6 ± 0.3

Albumin (g/dl)	IP	4.7 ± 0.3	4.7 ± 0.2	4.7 ± 0.2	4.7 ± 0.2	4.7 ± 0.3
	VM	4.6 ± 0.2	4.7 ± 0.2	4.7 ± 0.2	4.6 ± 0.2	4.6 ± 0.2
	PL	4.6 ± 0.2	4.7 ± 0.2	4.6 ± 0.3	4.6 ± 0.2	4.5 ± 0.3

Lactate Dehydrogenase (U/l)	IP	185 ± 23	179 ± 26	189 ± 36	200 ± 32	189 ± 34
	VM	180 ± 30	172 ± 35	184 ± 78	179 ± 37	184 ± 34
	PL	191 ± 24	186 ± 32	180 ± 29	191 ± 28	197 ± 26

Creatine kinase (U/l)	IP	124 ± 78	126 ± 70	149 ± 192	152 ± 113	141 ± 107
	VM	118 ± 89	139 ± 114	657 ± 2556	134 ± 75	127 ± 112
	PL	163 ± 225	140 ± 212	112 ± 83	126 ± 89	197 ± 379

Amylase (U/l)	IP	31 ± 8	31 ± 9	30 ± 9	30 ± 8	30 ± 8
	VM	35 ± 11	35 ± 12	34 ± 10	33 ± 11	34 ± 11
	PL	35 ± 9	34 ± 8	34 ± 8	33 ± 7	32 ± 8

Glucose (mg/dl)	IP	89 ± 18	81 ± 12*	84 ± 14	82 ± 12	83 ± 13
	VM	89 ± 16	82 ± 10	83 ± 9	85 ± 11	85 ± 16
	PL	81 ± 10	89 ± 18	86 ± 19	85 ± 20	89 ± 15

Cholesterol (mg/dl)	IP	178 ± 27	192 ± 26	177 ± 26	176 ± 26	192 ± 29
	VM	178 ± 25	194 ± 32	183 ± 29	185 ± 28	193 ± 26
	PL	191 ± 44	200 ± 48	194 ± 48	192 ± 45	203 ± 43

Triglycerides (mg/dl)	IP	116 ± 86	117 ± 68	108 ± 71	121 ± 101	109 ± 73
	VM	86 ± 33	94 ± 38	87 ± 46	87 ± 40	86 ± 32
	PL	123 ± 65	120 ± 52	121 ± 56	110 ± 48	124 ± 66

Thromboplastin time (sec)	IP	30 ± 3	nd	nd	nd	30 ± 3
	VM	30 ± 3	nd	nd	nd	30 ± 3
	PL	31 ± 3	nd	Nd	nd	30 ± 4

Prothrombin time (%)	IP	106 ± 13	nd	nd	nd	105 ± 11*
	VM	108 ± 12	nd	nd	nd	108 ± 10
	PL	107 ± 11	nd	nd	nd	109 ± 11

IP = Iscucin^® ^Populi, VM = Viscum Mali e planta tota, PL = placebo, nd = not done.

* p < 0.05 compared to placebo, ** p < 0.01 compared to placebo. Significant deviations are bold typed.

The only group mean outside the reference values was the creatine kinase level in the VM group at week 8 with a group mean of 657 U/l. It was caused by one very high value of 11811 U/l from a subject who trained mountain biking the day before. The subsequent CK-level under continued application of the study medication after 12 weeks was normal (118 U/l). The second highest CK-value lay with 254 U/l outside the reference values, too, but the third highest value lay inside the reference range (154 U/l).

With regards to the logarithmic values of alkaline phosphatase a difference could be shown, between the IP and the placebo groups at week 12 (p = 0.023) meaning that the value in the IP group was a little higher in comparison to the placebo group. The difference is related to a decrease of this parameter in the placebo group and not to an increase in the IP group. The values at baseline were not different to values after 12 weeks treatment or follow up in the IP group. All the values were well within the normal range (upper limit of normal = 129 U/l, highest value measured = 126 U/l).

With respect to blood glucose differences could be shown between the IP and the placebo group after 4 weeks treatment (p = 0.040). The mean values were smaller in the IP group. Four values out of 29 values in the IP group were below the lower limit of normal (70 mg/dl), the lowest being 48 mg/dl. In comparison, in the placebo group, two of 20 values were below the reference value after 4 weeks treatment, with a minimum value of 68 mg/dl. None of the low glucose concentrations was accompanied by clinical symptoms. Therefore, the low glucose concentrations are most likely related to a delayed sample analyses, because it is well known that glucose concentrations rapidly decrease in blood samples (6 mg/dl per hour at room temperature)[19].

After 4 weeks treatment, differences were seen between the VM and placebo group with regard to the natural logarithm of aspartate aminotransferase (ALT) levels (p = 0.007). The values were smaller in the VM group. Lower ALT values have no clinical relevance.

Prothrombin time showed a decrease in the IP compared to the placebo group at follow up (p = 0.026), but all values were within normal limits. The difference is related to an increase of this parameter in the placebo group rather to a decrease in the IP group.

Total bilirubin showed a difference between the VM- and placebo group (p = 0.036) after 12 weeks treatment with higher values in the VM group. A total of 5 probands had intermittently or constantly elevated bilirubin levels (3 in the VM and 2 in the placebo group) up to a maximum of 1.9 mg/dl (normal range up to 1.2 mg/dl). These elevated levels are most likely related to subjects with Gilbert's syndrome. Gilbert's syndrome is a disturbance of bilirubin glucuronidation, which frequently (about 5% of the population), causes fluctuating bilirubin levels and has no clinical significance [20]. Clinical examinations and other parameters of the liver function (prothrombin time) and integrity of the liver cell (ALT, AST) are normal in subjects with Gilbert's syndrome and were normal in our subjects with isolated elevation of the bilirubin level. Furthermore, at baseline, bilirubin levels in all of these 5 subjects were already close to or slightly above the upper limit of normal without any clinical or other laboratory sign of liver disease.

After 12 weeks treatment differences of platelet count were observed between the IP and the placebo group (p = 0.034) and between the VM and the placebo group (p = 0.014) with smaller values in the IP and VM group. However, there was no systematic change and values also strongly fluctuated in the placebo group.

## Discussion

The placebo-controlled, 3-armed modified phase 1 trial presented here was planned and conducted according to all current standards. Randomization was concealed and successful: the groups were well balanced at baseline. The number of missing data is very low: all but one proband completed the study regularly. All parameters were analyzed and reported as planned in the study protocol. There was, therefore, no risk of bias regarding sequence generation, allocation, incomplete or selective outcome reporting. Known from the literature, partial deblinding occurred, because local reactions indicated treatment with one of the mistletoe preparations [21]. However, the study remained partially blinded, because not all of the individuals receiving Viscum Mali e planta tota (VM) or Iscucin^® ^Populi (IP) developed local reactions. Furthermore, all analyses of safety laboratory and immunological parameters were performed blinded. Therefore, partial deblinding most likely did not affect the results.

The primary aim of the study was to investigate for the first time safety and tolerability of the two mistletoe preparations IP and VM in humans to fulfil the requirements of the German regulatory authority (BfArM). Therefore, the preparations were injected in a dose escalating manner.

In order to achieve high internal validity the study was performed in healthy subjects. Whether or not these data are transferable to tumor patients is a matter of debate. Because tumor patients are confronted with a life threatening disease and often experience severe side effects from conventional therapy, it can be speculated that tolerance to harmless local reactions at the site of injection is higher than in healthy individuals. This well known side effect of IP which is also mentioned in the Investigator's Brochure [15] and in a review about other mistletoe preparations [22] probably plays a lesser role in clinical practice than suggested from our study with healthy subjects. At least the strong local reactions and frequency of dose adjustments in our study show, that we used the maximum tolerable dose and that the data are not biased by under-dosing.

Our data show, that IL-6 serum levels did not increase after subcutaneous injection of the mistletoe preparations IP and VM. IL-6 was measured with highly sensitive methods and under strict adherence to the pre-analytic requirements [19]. It was measured in parallel to the other immunological parameters and in parallel to the occurrence of local reactions at the injection site. Therefore, the results can be regarded as valid, despite the short half-life of IL-6 in blood. This is supported by the fact that CRP, which is induced by IL-6 in the liver and has a longer half-life of 19 h, was also not increased during application of the mistletoe preparations. Even if we would have missed a temporary systemic increase of IL-6, CRP would have been reacted.

In multiple myeloma patients IL-6 is often synthesized by the tumor itself and by bone marrow stem cells within an autocrine growth loop [23]. We of course cannot rule out, that mistletoe preparations interfere in this loop, because we investigated healthy probands. But we can at least conclude that no substantial amounts of IL-6 are produced from the normal immune system after application of different dosages IP or VM.

IP strength H has a high mistletoe lectin (ML) content of about 5300 ng/ml [14]. Because ML are responsible for the most dominant immunological effects of mistletoe preparations in vivo [18], our results are most likely also transferable to other mistletoe preparations with related ML-content. Other smaller studies investigating other mistletoe preparations are in accordance with our findings ([24-26], for review see [27]) For example in 8 breast cancer patients 10 weeks subcutaneous treatment with 0.2 mg/30 kg of the mistletoe preparation AbnobaViscum Mali (approximately 75 ng/ml/30 kg ML) did not result in systematic changes of IL-6 concentration in supernatants of un-stimulated and stimulated peripheral mononuclear cells before and during treatment compared to baseline [25]. In only one small clinical study presenting 4 not further described individuals [28] in part increasing IL-6 levels after single injections of mistletoe extract have been described. However, due to the poor reporting quality and selection, the validity of this report is limited.

Interestingly, despite the absence of IL-6 stimulation, we found significant immunological changes during treatment with IP, namely a dose dependent, distinct increase of eosinophils. Eosinophilia during mistletoe therapy has been described before and is related to the content of ML in the extracts [16,18,29]. However, a mean tenfold increase as observed in the present study has not yet been reported in a clinical study. Because eosinophils are stimulated by T-helper-2 cells, which produce IL-5 and simultaneously stimulate B-cells [30], it is plausible that the number of CD4 cells and IgM were increased in the IP group.

Eosinophils have cytotoxic properties and may play a role in the host response against cancer [31,32]. There exists evidence from epidemiological studies, that eosinophilia correlates with a better prognosis in a variety of cancer types ([33,34] for review see [32]). Whether or not eosinophilia has a clinical benefit for cancer patients during treatment with IP requires further investigation. VM caused only a mild increase of eosinophils in some individuals treated with strength D2 and 2%.

In order not to miss a potential risk, a comprehensive safety analysis was performed. Several group differences were found between the two mistletoe preparations and placebo and single values were outside the reference range. However, conspicuous values could be explained by reasons unrelated to the study medication, e.g. elevated creatine kinase levels after exercise, low glucose due to transport time and intermittent elevations of bilirubin due to Gilbert's syndrome, which has no clinical significance. At single visits, C-reactive protein and alanine aminotransferase (ALT) levels were in the mistletoe groups significantly different from placebo, however, the values were higher in the placebo group. Prothrombin time and platelet count were significantly lower in the IP or VM group at single visits. Because values remained within normal limits and no systematic changes were found, the differences are most likely related to multiple testing. Performing 100 statistical comparisons on a significance level of 5% automatically generates a risk of 5 false results. For the parameters in table 6 alone we performed 188 statistical comparisons (25 laboratory parameters, except prothrobin time and thromboplastin time each tested at 4 time points for the two mistletoe preparations versus placebo), resulting in a probability of 9 false results. We had exactly 9 significant group differences at single visits regarding the parameters in table 6, which is in the expected range. In addition to significant group differences we carefully searched for systematic deviations and values outside the normal range but also this strategy did not reveal a potential risk of the two mistletoe preparations. Because our analysis revealed no hint for side effects of the tested preparations on liver, kidney, respiratory, gastrointestinal, cardiovascular and nervous system and no suppressive effects on bone marrow- or immune functions could be found, the risk of side effects on inner organs is therefore most likely to be negligible in tumor patients, too.

Nevertheless, local reactions from application of IP strengths G and H can be strong. Therefore, a whole ampoule of IP strength G or H should preferably only be used in patients who have been pre-treated with lower doses of IP to induce tolerance. Local reactions are related to the preparation's mistletoe lectin (ML) content. This relationship has been shown in other studies and declines with prolonged treatment and development of ML-antibodies [16,18]. In the present study, ML-antibodies also formed in the IP-treated subjects and local reactions (week 6 = 8.9 cm) declined with prolonged treatment (week 12 = 6.2 cm) although the amount of injected mistletoe extract was increased. High doses of other mistletoe preparations causing similar strong local reactions have been clinically used with beneficial effects for the treatment of hepatocellular carcinoma with beneficial effects [35]. These data provide justification for further investigation of the effects of highly concentrated mistletoe preparations. VM does not cause strong local reactions and was very well tolerated at all dose levels.

## Conclusion

Iscucin^® ^Populi is a strong stimulator of eosinophils, probably generated via a T-helper-2 cell activation. It does not have immunosuppressive effects or stimulate markers of inflammation like C-reactive protein or IL-6 production. Viscum Mali e planta tota promotes only a mild increase of eosinophils and is well tolerable.

## Competing interests

None for RH, HL, JW. CB is employee of WALA Heilmittel GmbH which sponsored the study

## Authors' contributions

RH planned the study design and conducted the study, HL performed the statistical planning and analyses of the study and wrote the final report, JW was substantially involved in data acquisition and performing of the study, CB organized all the legal and administrative requirements to perform the study and contributed to the coordination of the study. All authors read and approved the final manuscript.

## Pre-publication history

The pre-publication history for this paper can be accessed here:

http://www.biomedcentral.com/1472-6882/11/116/prepub
